# Thrombophlebitis in an Adolescent Patient With Primary Carnitine Deficiency: A Case Report

**DOI:** 10.7759/cureus.117164

**Published:** 2026-09-29

**Authors:** Douaa Kaouani, Noama Ryad, Mariam Lagrine, Sara Ouassil, Hussein Choukri Ahmanna, Rabiy Elqadiry, Houda Nassih, Aicha Abourrahouat, Hicham Jalal, Imane Aitsab

**Affiliations:** 1 Department of Pediatrics, Mohammed VI University Hospital Center, Cadi Ayyad University, Marrakech, MAR; 2 Department of Radiology, Mohammed VI University Hospital Center, Cadi Ayyad University, Marrakech, MAR

**Keywords:** inferior vena cava agenesis, l-carnitine, primary carnitine deficiency, superficial thrombophlebitis, trimethylamine n-oxide, venous thrombosis

## Abstract

Primary carnitine deficiency (PCD) is a rare autosomal recessive disorder caused by biallelic pathogenic variants in SLC22A5, leading to defective carnitine transport and impaired mitochondrial long-chain fatty acid β-oxidation. Lifelong L-carnitine supplementation constitutes the mainstay of therapy and has been shown to effectively prevent metabolic decompensations. However, oral carnitine is partially metabolized by gut microbiota to trimethylamine, which is subsequently converted to trimethylamine N-oxide (TMAO), a metabolite that has been associated with increased thrombotic risk in both experimental and observational studies. We report the case of a 14-year-old boy with PCD on long-term, high-dose L-carnitine who developed acute superficial thrombophlebitis of the left great saphenous vein in the presence of previously undiagnosed congenital infrarenal inferior vena cava (IVC) agenesis with extensive collateral varices. No conventional prothrombotic risk factors were identified. The patient was successfully treated with anticoagulation while L-carnitine therapy was continued. This case illustrates that congenital IVC anomalies may predispose patients with PCD to venous thrombosis and raises the hypothesis that chronic L-carnitine supplementation may contribute as an additional prothrombotic factor through TMAO elevation.

## Introduction

Primary carnitine deficiency (PCD), also known as carnitine uptake defect, is an autosomal recessive inborn error of metabolism caused by biallelic pathogenic variants in the SLC22A5 gene encoding the OCTN2 carnitine transporter [1-3]. Deficient carnitine transport results in urinary carnitine wasting, intracellular carnitine depletion, and impaired mitochondrial β-oxidation of long-chain fatty acids [4,5]. Clinical manifestations range from life-threatening hypoketotic hypoglycemia, cardiomyopathy, and encephalopathy in infancy to later-onset myopathy or cardiomyopathy [6].

Newborn screening has enabled early diagnosis and presymptomatic treatment with oral L-carnitine (typically 50-100 mg/kg/day), which is highly effective at preventing acute metabolic decompensations and improving cardiac and muscular function [5,7].

A portion of ingested L-carnitine escapes small-intestinal absorption and reaches the colon, where gut microbiota metabolize it to trimethylamine (TMA) [8]. TMA is absorbed and oxidized in the liver by flavin-containing monooxygenase 3 to trimethylamine N-oxide (TMAO) [8]. Elevated TMAO levels have been associated with increased platelet reactivity, endothelial dysfunction, and thrombotic risk [9,10].

Venous thrombotic events are uncommon in PCD. When reported, they have usually been attributed to cardiomyopathy or acute metabolic decompensation rather than therapy [6]. Congenital anomalies of the inferior vena cava (IVC), including infrarenal agenesis, are rare but well-recognized causes of lower-extremity venous thrombosis due to chronic venous stasis and collateral vessel formation [11,12].

Here we describe a pediatric patient with genetically confirmed PCD on stable long-term L-carnitine therapy who developed superficial thrombophlebitis in the setting of previously unrecognized infrarenal IVC agenesis.

## Case presentation

A 14-year-old Moroccan boy, born to consanguineous parents with a family history of sibling death from suspected metabolic disease, was diagnosed with PCD at 15 months of age following an episode of hypoketotic hypoglycemia and encephalopathy during a viral illness. Genetic testing confirmed homozygous SLC22A5 mutations. He remained metabolically stable for the subsequent 13 years on oral L-carnitine supplementation at 100 mg/kg/day (maximum 3 g/day), with consistently therapeutic plasma carnitine levels and no further decompensations.

At presentation, he reported a one-week history of progressive left thigh pain, swelling, and localized warmth without fever or overlying erythema. He was afebrile and hemodynamically stable on admission. Physical examination demonstrated a warm, tender swelling of the left thigh with a positive Homans’ sign and reduced calf compressibility. No clinical features suggestive of infection, recent immobilization, or conventional prothrombotic risk factors were identified.

Laboratory investigations revealed elevated D-dimer and C-reactive protein levels. A comprehensive thrombophilia work-up including Factor V Leiden mutation, prothrombin G20210A mutation, protein C and protein S activity, antithrombin III level, antiphospholipid antibodies (lupus anticoagulant, anti-cardiolipin, and anti-β2-glycoprotein I), and plasma homocysteine was entirely negative. The complete blood count and standard coagulation profile were normal. Plasma carnitine concentrations remained within the therapeutic range.

Prior abdominal and pelvic CT imaging strikingly demonstrated congenital agenesis of the infra-renal IVC with dramatic compensatory collateral circulation, including marked dilatation of the left gonadal vein (both intra- and retroperitoneal segments) and extensive superficial parietal abdominal wall varices (Figures 1-3). Doppler ultrasound performed at presentation showed non-compressible segments of the left great saphenous vein filled with hyperechoic endoluminal material and absent color flow, without extension into the deep venous system, consistent with acute superficial thrombophlebitis. The patient was promptly initiated on subcutaneous enoxaparin for three days with anti-Xa and platelet monitoring, followed by oral rivaroxaban for three months. L-carnitine supplementation was maintained uninterrupted. Symptoms resolved completely within one week. Follow-up Doppler ultrasound at one month demonstrated full recanalization of the previously thrombosed segment, normal compressibility, and restored color flow (Figure 4).

**Figure 1:**
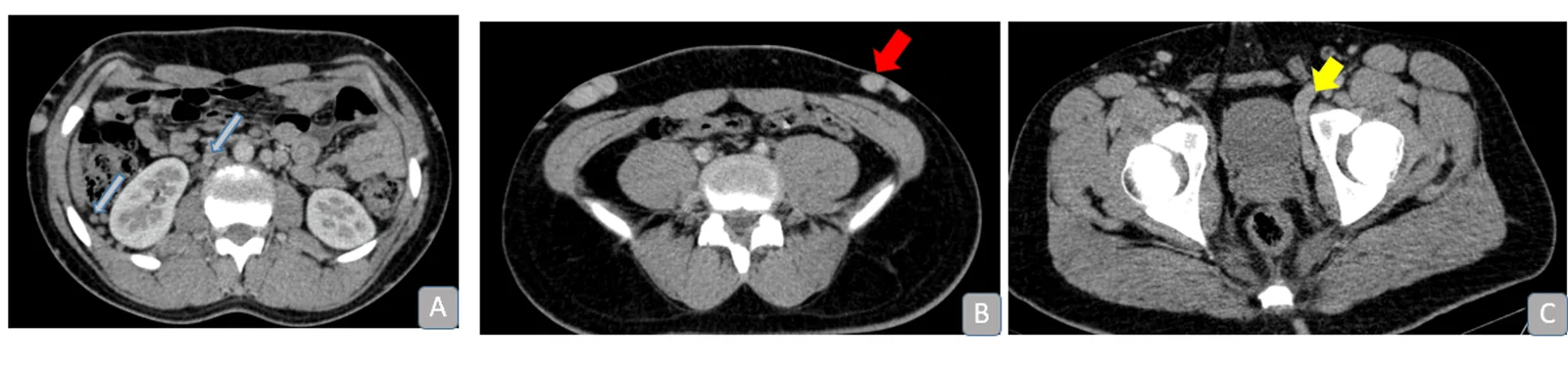
Abdomen-pelvis CT scan: axial slices after contrast injection: Dilation of the left gonadal vein (yellow arrow), intra- and retroperitoneal (blue arrows) and of the abdominal wall (red arrow).

**Figure 2:**
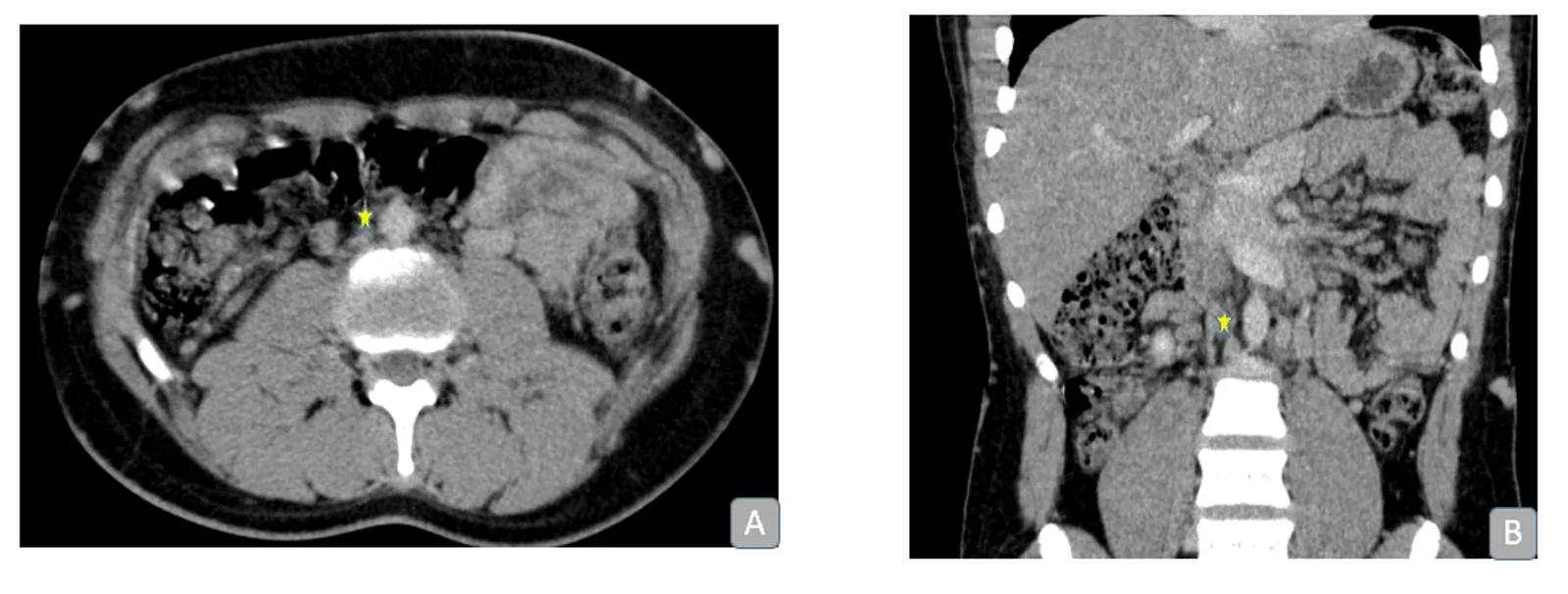
Axial (A) and coronal (B) views after contrast injection: absence of visualization of the infrarenal IVC (yellow stars). IVC: Inferior vena cava

**Figure 3:**
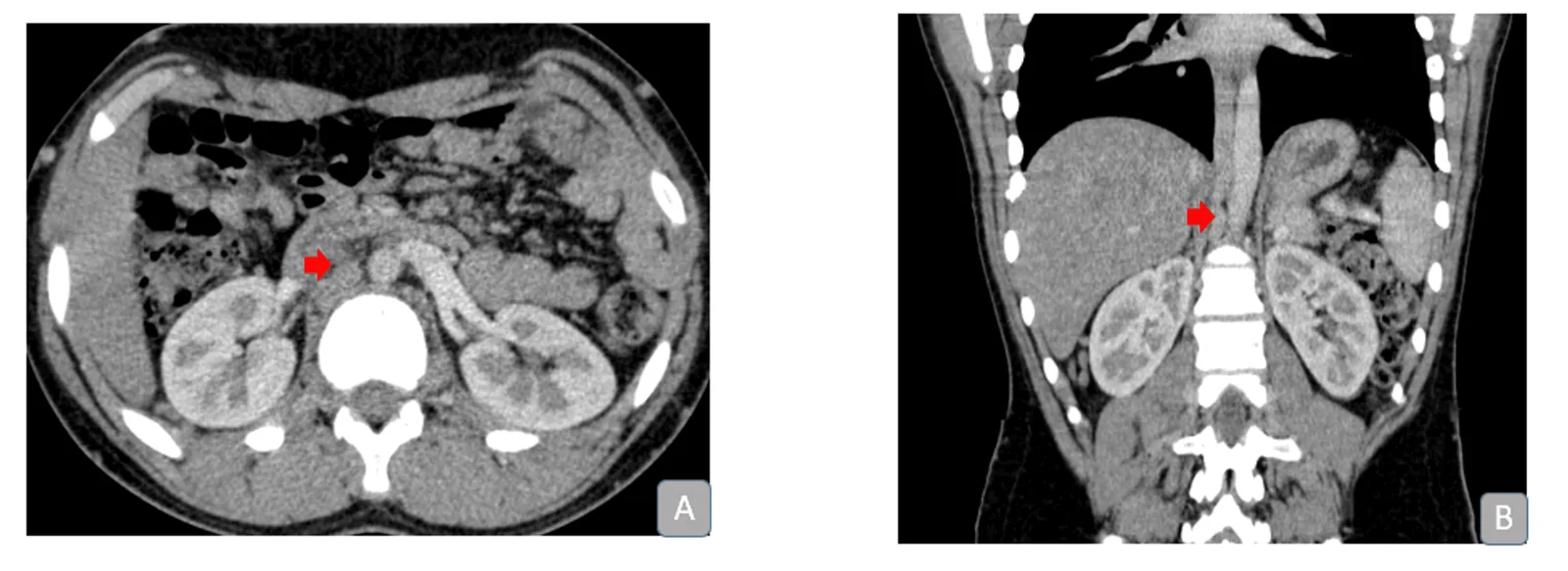
Axial (A) and coronal (B) views after contrast injection: presence of the supra-renal IVC (red arrows). IVC: Inferior vena cava

**Figure 4:**
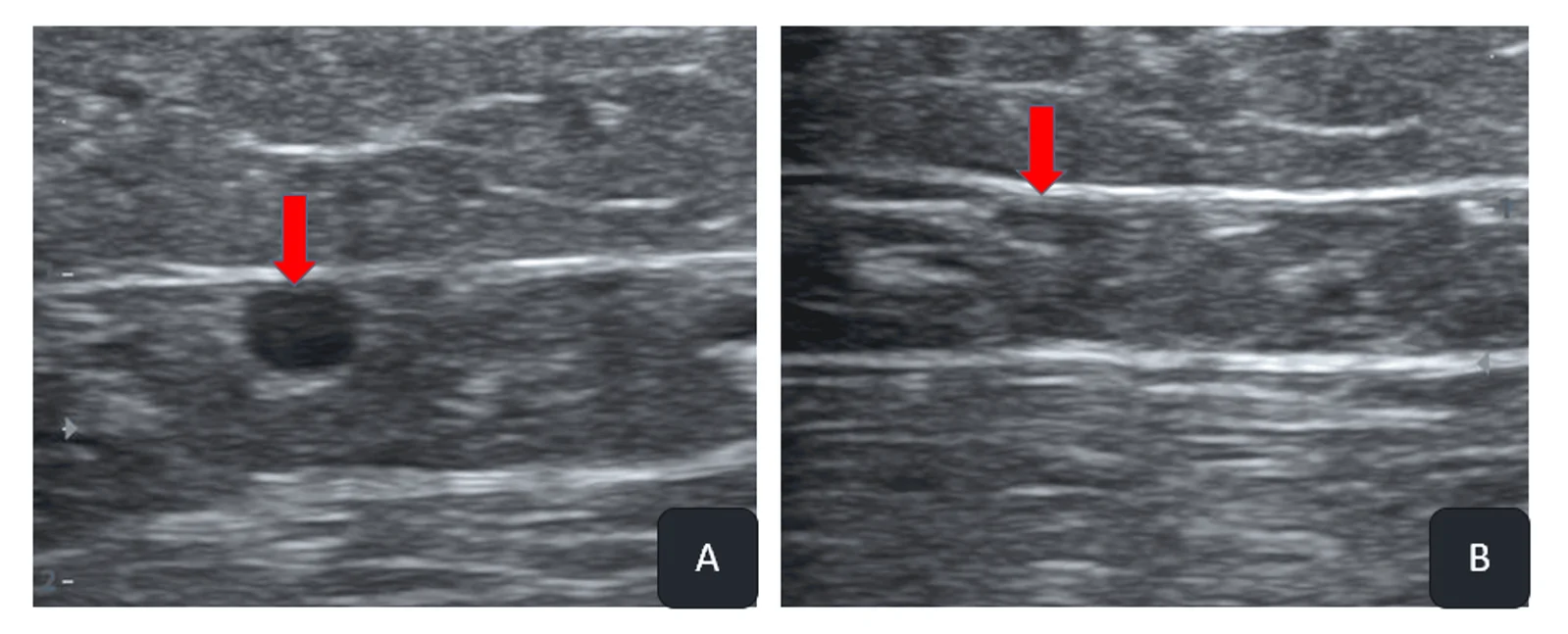
Follow-up Doppler ultrasound of the left internal saphenous vein (arrow) open (A) and compressible (B), confirming complete resolution of thrombophlebitis.

## Discussion

This case involves a 14-year-old boy with PCD who developed acute superficial thrombophlebitis of the left great saphenous vein after 13 years of stable high-dose L-carnitine treatment. Imaging revealed previously unrecognized congenital agenesis of the infrarenal IVC with extensive collateral vessels. Congenital IVC anomalies are a recognized cause of lower-extremity venous thrombosis because they create chronic stasis and turbulent flow in the collateral circulation [11,12]. In this patient, the thrombus formed precisely in a markedly dilated great saphenous vein that was serving as a major collateral, which strongly points to the anatomic abnormality as the main driver of the event.

Venous thrombosis is uncommon in PCD. When it has been reported, it has usually occurred in the setting of cardiomyopathy or acute metabolic decompensation [6]. The present case is different: the patient was metabolically stable, had no evidence of cardiomyopathy, and lacked conventional prothrombotic risk factors.

Oral L-carnitine is known to be metabolized by gut bacteria to TMA, which the liver then converts to TMAO [8,13]. Elevated TMAO levels have been linked in experimental work to increased platelet reactivity and endothelial dysfunction, and observational studies have associated higher TMAO concentrations with thrombotic events [9,10,14-17]. Patients with PCD take high doses of carnitine for life, so the theoretical possibility that TMAO could add to thrombotic risk when an anatomic predisposition is already present is worth considering. In this particular patient, however, TMAO was never measured, so any contribution remains purely speculative.

L-carnitine replacement is still the essential treatment for PCD and should not be stopped on the basis of this single report [5,7].

The case does highlight the value of looking for congenital venous anomalies when a patient with PCD develops unexplained venous thrombosis. Larger studies that combine serial TMAO measurements with systematic vascular imaging would be needed to determine whether long-term carnitine therapy meaningfully interacts with anatomic risk factors.

## Conclusions

This case describes acute superficial thrombophlebitis of the left great saphenous vein in an adolescent with PCD who had received long-term, high-dose L-carnitine supplementation. The thrombosis occurred in the setting of previously unrecognized congenital infrarenal IVC agenesis with extensive collateral varices. The anatomic abnormality provides a plausible explanation for the event through chronic venous stasis. Whether chronic L-carnitine therapy and possible TMAO elevation played an additional role remains speculative, as plasma TMAO was not measured in this patient.

The observation suggests that clinicians should consider evaluating for congenital venous anomalies when patients with PCD develop unexplained venous thrombosis. Further studies that include serial measurement of TMAO and systematic vascular imaging in larger cohorts of patients with PCD would be needed to clarify any potential interaction between long-term carnitine supplementation and anatomic risk factors for thrombosis.
